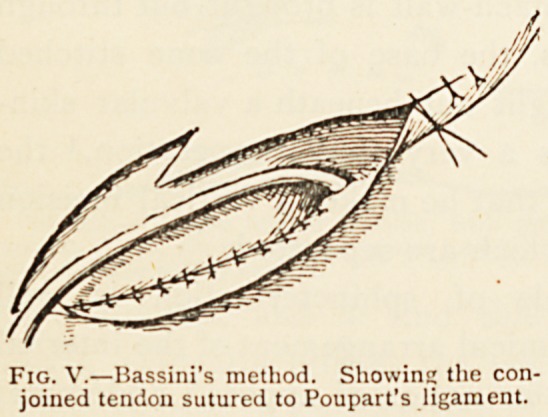# Abdominal Incisions

**Published:** 1900-09

**Authors:** Thomas Carwardine

**Affiliations:** Assistant Surgeon, Bristol Royal Infirmary


### ABDOMINAL INCISIONS.

BY

THOMAS CARWARDINE, M.S. (Lond.), F.R.C.S.,

*Assistant Surgeon, Bristol Royal Infirmary.*

---

THE abdominal parietes depend to a large extent for their arrangement upon the survival and evolution of those structures which are of use in the economy. We should therefore expect, as we find, that the normal anatomical arrangement of the abdominal wall, as the outcome of evolution, is the standard of perfection, the ideal of the wise surgeon.

In our reverie we may look back to the time when our bellies had ribs—to be tickled, or broken, or what not. In the wisdom of Nature these have departed, leaving as their vestiges certain tendinous intersections, constant in the recti and occasional in the internal oblique. In short, the belly is a chest which has undergone metamorphosis. Accordingly, the musculature of the abdomen is homologous with that of the thorax, and agrees with it in continuity of directions. Thus the fibres of the external oblique pass downwards and forwards like those of the external intercostals, and those of the internal oblique pass upwards and inwards like those of the internal intercostals. Together they form two decussating abdominal costal muscles. The third muscle, the transversalis, is continuous and homologous with the triangularis sterni. The whole form a muscular lattice-work of decussating fibres, strongest where the decussation is most pronounced, which braces up the parietes during every strain or cough. It is Nature's method of preventing ventral hernia, and it is only lately that surgeons have realised, in its imitation, the secrets of success.

Sphincter incisions by separation of muscular fibres, and the principle of valve-action whenever two musculo-aponeurotic layers are superimposed, are bound to dominate the surgery of the future; and as the writer has paid attention to this subject during operations on the abdomen, a few remarks may be useful.

Let me in the first place indicate that the transverse division of a muscle is a very serious affair. Be the suturing ever so perfect, muscular continuity is not re-established, but a yielding

FIG. I.—Diagram of the musculature of the abdomen, chiefly designed to illustrate the advantages of separation rather than division of muscular fibres. It will be observed that Langenbuch's incision divides nerves passing to the right rectus. *a*, Langenbuch's incision for exposing the right kidney; *b*, Region showing muscular lattice-work and direction of nerves; *c*, Sphincter incision for appendicitis; *d*, Bassini's incision; *e*, Sphincter incision for gastrostomy; *f*, Sphincter incision for exposing kidney; *g*, Sphincter incision for inguinal colostomy; *h*, Median cœliotomy.

scar of variable width results. The division of a muscle always involves permanent degeneration of muscular fibres to a certain distance on each side of the line of section. This is explicable by the fact that the intramuscular nerve-fibres of the locality degenerate, for they run some distance between the muscular fibres before they terminate. The author has often sutured divided muscle, but has invariably met with a measure of disappointment. It is also a serious matter to divide the nerve going to a muscle, for the result—atrophy—is the same as that from division of the muscle, except that the aponeurotic capsule and the epimysium remain intact.

The division of an aponeurosis is not so serious, but it is wisely avoided, for a scar is at best a poor substitute for a tendon; and if the aponeurosis ends in muscular fibres, the latter will either stretch the scar or degenerate from impaired function.

The judicious principle, therefore, in abdominal operations, is to incise in the direction of the fibres of the aponeurosis, to separate the fibres of that aponeurosis, and to separate the muscular fibres in the line of their direction—which is usually at an angle to that of the overlying aponeurosis. Let us see how these facts may be applied to present-day surgery.

**Appendicitis.**—The advantages of muscular separation for this condition, advocated by McBurney, are now recognised, so that a surgeon who continues to make a direct incision through the musculature is behind the times. When once a hernia has formed in the scar of a direct incision the difficulties of its cure are great, for, as explained above, the musculature has atrophied. But a hernia here is as easy to prevent as it is difficult to cure. One has only to make an incision in the line of the aponeurosis of the external oblique, to separate its fibres, and then to separate the combined fibres of the internal oblique and transversalis in a direction upwards and inwards, and we have a perfect sphincter-opening which does not really require any sutures afterwards. (Fig. I. *c*.) I find this incision adequate not only for the removal of the appendix in the quiescent interval, but also for the treatment of acute suppurating cases, and am convinced that there is no other method of incision equal to it.

Some have advocated an incision upwards and inwards towards the umbilicus; but this is open to two objections—first, that the aponeurosis of the external oblique is cut across; second, that the musculature of the combined internal oblique and transversalis is transverse at the level of the anterior superior spine, and not oblique.

An incision at the outer border of the rectus, with mesial deflection of that muscle, has been advocated by Battle,[1] Kammerer,[2] Jalaguier,[3] and Lennander.[4] It will be seen that this may involve division of one of the nerves to the rectus. But the resulting atrophy of the rectus is not constant, and when it occurs it appears to be recovered from in time. It will be remembered that the rectus is not normally segmented below the umbilicus, which probably explains the inconstancy of atrophy here.

**Colostomy.**—There can be no doubt that the method of Maydl-Reclus produces the best results. In this the musculo-aponeurotic structures are merely separated in the directions of their fibres (Fig. I. *g*), and a loop of gut brought out and fixed without sutures by means of a glass rod through its mesentery. The spur is perfect, effectually preventing any passage of fæces from the upper to the lower bowel, and the sphincter is so excellent that a large receptacle is unnecessary and the patient can defæcate at decent intervals. Fig. II. shows such a colostomy in section.

FIG. II.—Section of colostomy opening.

The principle of muscular separation is gradually becoming adopted. Thus H. Allingham, who formerly operated by direct incision through muscles, now separates the musculature.

**Nephrotomy.**—Except by very long incisions, the kidney cannot be freely exposed by separation of musculo-aponeurotic structures alone. But I have several times exposed it by this means, and with slight division of musculature have been able to bring the kidney out on to the loin. The lumbar skin-incision must be a long one in the direction of the fibres of the

[1] *Brit. M. J.*, 1895, ii. 1360.

[4] *Centralbl. f. Chir.*, 1898, xxv. 90.

external oblique (Fig. III.), and the separation of the internal oblique must be commenced well forwards, the exposure of the kidney becoming almost abdominal rather than lumbar. (Fig. I. *f*.) The method has been spoken of highly by Mayo Robson, and I find that my former master, Henry Morris, evidently utilises the principle, for he now makes an oblique incision in the direction of the external oblique muscle.[1] Appreciating the principles, and frequently operating on the kidney, one could not do otherwise than fall into the habit of separation.

Fig. III.—Oblique lumbar incision, with separation of muscular fibres, showing fibres of the internal oblique exposed with the kidney beyond.

Ventral nephrectomy by Langenbuch's incision (Fig. I. *a*) has the disadvantage of dividing nerve fibres to the rectus. A long oblique lumbar incision, with separation of fibres of the internal oblique anteriorly, is to my mind preferable.

**Gall-bladder and Bile-ducts.**—Although the after-tendency to hernia is less in the hypochondriac regions than elsewhere, attempts are now made to incise on the sphincter principle by separation of the outer fibres of the rectus or else of the lateral abdominal muscles. A. D. Bevan[2] has proposed an *f*-shaped incision, with transverse extensions above and below, if necessary, so as to diminish risk of division of nerves. My own predilection is for an oblique incision upwards and inwards in the line of the internal oblique, with separation of its fibres, and continued horizontally above—like Γ opened out.

**Median Cœliotomy.**—In order to prevent hernia some surgeons make the incision about 1 inch to the right or left of the middle line and separate the muscular fibres of the rectus before opening the peritoneum, which is thus exposed by a sphincter-incision.

**Gastrostomy.**—It was an early improvement on Howse's original operation to separate the muscular fibres of the rectus

[1] *Renal Surgery*, 1898, p. 117. [2] *Ann. Surg.*, 1899, xxx. 15.

and draw the stomach through the sphincter so formed, and this principle has been employed in all the methods since introduced. (Fig. I. *c*.) The combination of sphincter and valve is especially brought out in Frank's method of gastrostomy, in which a cone of stomach-wall is brought out through separated fibres of the rectus, the base of the cone stitched there, and then the apex brought out beneath a valvular skin-flap. Mayo Robson performs a very similar operation,[1] the only essential difference being that he makes a vertical incision over the rectus, the fibres of which are separated.

**Herniotomy.**—The principle of sphincter-action is well illustrated in the normal anatomical arrangement of the internal abdominal ring, and the valvular action of apposed abdominal planes is borne out in the marked success which has followed Bassini's operation for the radical cure of hernia.

*The Sphincter.*—I am not aware that surgical attention has before been clearly drawn to the anatomical sphincter of the internal ring, which I will now describe. If the ring be examined, it will be found that certain muscular fibres pass from the outer third of Poupart's ligament into the conjoined tendon (Fig. IV. *c*), which I venture to call the tensor muscle of the conjoined tendon. It is obvious that whenever a man coughs or strains this muscle contracts and straightens the upper arch over the internal ring. Again, certain muscular fibres of the external oblique are inserted into Poupart's ligament between the lines *c* and *d* (Fig. IV.). On straining, these fibres would pull Poupart's ligament up and

FIG. IV.—To illustrate the inguinal sphincter and valve. *a*, Internal ring; *b*, External ring; *c*, Tensor muscle of the conjoined tendon; *d*, Conjoined tendon. Above *c* are the tensor fibres of the external oblique.

[1] *Brit. M. J.*, 1900, i. 697.

straighten the lower arch of the internal ring. The two sets of fibres act conjointly as a true sphincter to the internal ring. The only modification of this sphincter in herniotomy worth noting is that in Bassini's operation the cord is held out of the way, and the inner part of the upper arch is sutured to the lower arch, thus displacing the cord outwards, and at the same time greatly curtailing the aperture between the two halves of the sphincter and approximating their insertions. (Fig. V.)

FIG. V.—Bassini's method. Showing the conjoined tendon sutured to Poupart's ligament.

*The Valve.*—The sphincter aforesaid is reinforced in its action by what we may term the great inguinal valve—closed by the apposition of the anterior and posterior walls of the canal during every expulsive effort (Fig. IV.). The effectiveness of this valve must depend upon the length of it, *i.e.* upon the length of the inguinal canal.

Now all operations for the radical cure of hernia, with one exception, tend to shorten this valve. The exception is Bassini's operation, by which the external oblique is slit up, the aperture of the inguinal sphincter diminished, and the length of the great inguinal valve markedly increased. When the conditions for Bassini's operation are favourable, that operation is to my mind by far the most reliable method we have for the radical cure of inguinal hernia. The resulting firmness of the abdominal wall is not approached by any other method. The absolute essential is asepsis. If suppuration should occur, the results may be disastrous. But with due care, suppuration may be almost certainly avoided, and I have used as many as thirty sutures in the operation without the least sign of infection. It is, to my mind, the only operation for radical cure which is rationally based upon Nature's plan of muscular sphincter and aponeurotic valve.

Certain American surgeons have gone to the extreme of dividing the posterior wall of the inguinal canal, and transplanting the cord inwards. One surgeon has the audacity to advise passing the cord through a sort of button-hole made in the os pubis. Such procedures are more the result of inordinate mechanical originality than the product of a wise application of the lessons which Nature affords. The surgeon may take it for granted that the mechanical devices wrought by evolution are sounder than the machinations of his own imagination.

---

### SPINDLE-CELLED SARCOMA OF THE HARD PALATE.

## Figures and Tables

**Fig. I. f1:**
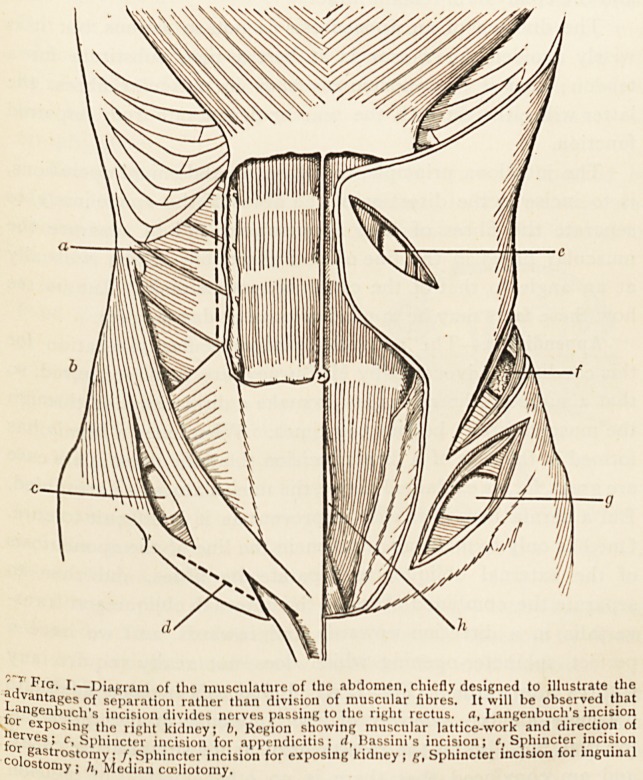


**Fig. II. f2:**
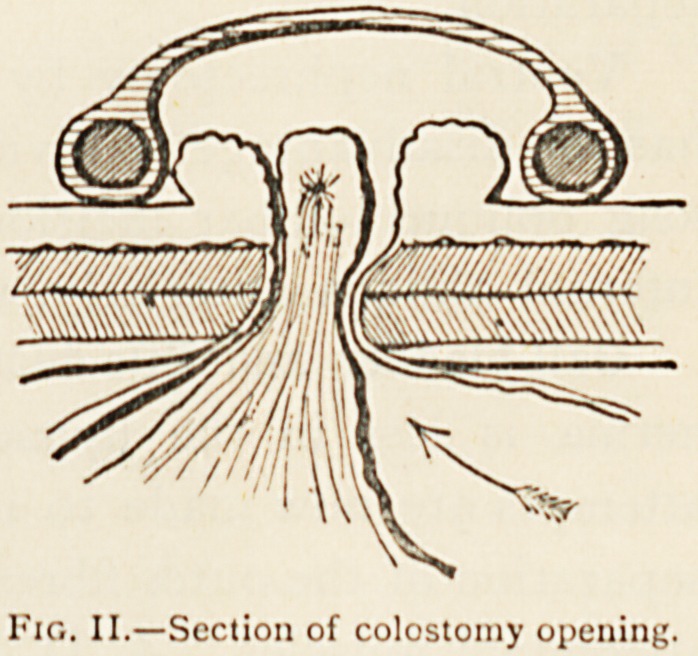


**Fig. III. f3:**
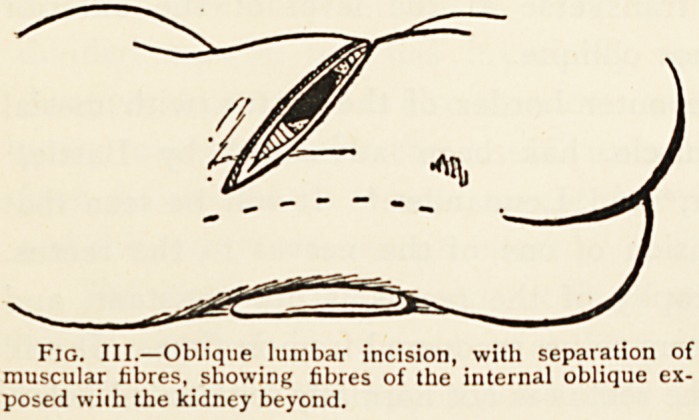


**Fig. IV. f4:**
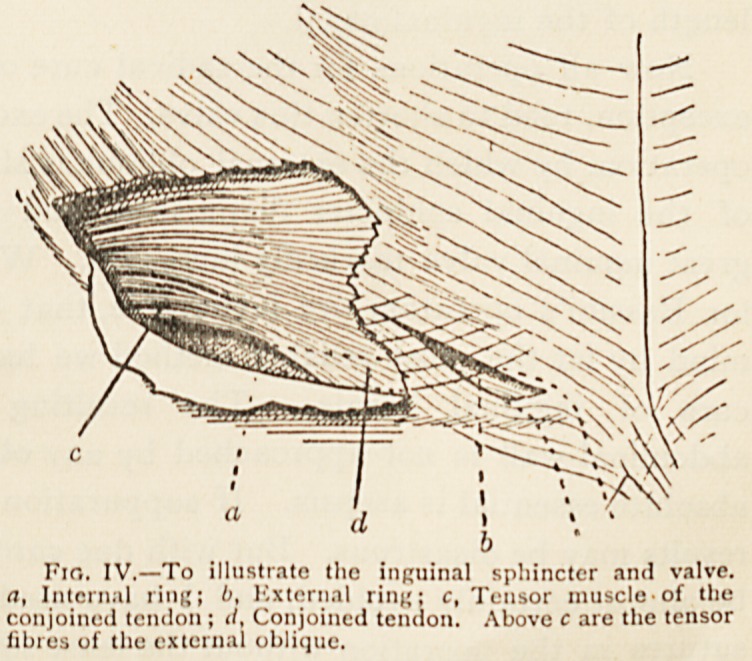


**Fig. V. f5:**